# Are We Ready With Prevention for Type 1 Diabetes?

**DOI:** 10.1002/dmrr.70101

**Published:** 2025-10-25

**Authors:** Evelina Maines, Roberto Franceschi, Francesca di Candia, Enza Mozzillo

**Affiliations:** ^1^ Department of Pediatrics S. Chiara Hospital of Trento APSS Trento Italy; ^2^ CISMed—Centro Interdipartimentale di Scienze Mediche University of Trento Trento Italy; ^3^ Department of Translational Medical Science University of Naples Federico II Naples Italy

**Keywords:** children, prevention, review, screening, type 1 diabetes

## Abstract

Definitive prevention for type 1 diabetes (T1D) is not yet available, but we are now entering a new era where disease‐modifying therapies are becoming available in T1D to halt disease progression. In this review, we present up‐to‐date knowledge related to the prevention of T1D in youth, involving disease‐modifying therapies at different T1D stages. A narrative literature review utilising the PubMed/MEDLINE database was performed using the keywords ‘screening’, ‘prevention’, ‘Disease‐modifying therapy’, and ‘Diabetes Mellitus, Type 1’ [Mesh] in youth aged 0–18 years. Only teplizumab has been approved by the FDA as the first drug shown to delay the onset of Stage 3 T1D. Other monoclonal antibodies targeting specific immune cells, agents targeting specific cytokines, antigen‐specific therapies, and immunomodulant and/or immunosuppressive agents have been studied alone or in combination to control or delay the progression of beta‐cell destruction. In individuals with Stage 3 T1D, several intervention trials have led to a temporary improvement in beta‐cell function, but this benefit has consistently been short‐lived. Ongoing and future research will be essential to refine patient selection, identify additional therapeutic targets, and optimise the timing and durability of immunotherapy responses.

## 1 Introduction

Type 1 diabetes (T1D) is an autoimmune disease characterised by permanent hyperglycemia and insulin deficiency. Four major islet autoantibodies (IAbs) are the immune biomarkers: autoantibodies against insulin (IAA), glutamic acid decarboxylase‐65 (GADA), insulinoma‐associated antigen‐2 (IA‐2A), and zinc transporter 8 (ZnT8‐A). T1D results from complex interactions between the immune system, genes, and the environment. Eisenbarth et al. [1] proposed a model of the natural history of T1D involving the presence of genetic risk and environmental factors that act as triggers and initiate autoimmune destruction of beta‐cell mass in function of age.

A novel classification of T1D, recently proposed by Insel et al. [2], includes the concept of distinct identifiable stages of T1D: Stage 1, presymptomatic with at least 2 IAbs+ and normoglycaemia; Stage 2, presymptomatic with at least 2 IAbs+ and dysglycaemia; Stage 3 clinical onset of T1D with hyperglycaemia that may be symptomatic or not. Dysglycaemia is defined as impaired fasting glucose (100–125 mg/dL), impaired glucose tolerance (140–199 mg/dL at the 2‐h oral glucose tolerance), abnormal glycated haemoglobin A_1c_ (HbA_1c_) (5.7%–6.4%), or an increase in HbA_1c_ by 10% from the prior measure [2]. Children in Stage 2 and Stage 3 T1D have to be followed up with repeated IAb testing and glucose level monitoring to identify those who progress to Stage 3 T1D. The presence of a single autoantibody or a transient single autoantibody represents the presymptomatic early stage 1 of T1D (pre‐stage 1 T1D) [3]. Therefore, even positivity for a single IAb, although not nominally classified as a pre‐symptomatic stage, is associated with the risk of progression to T1D [4].

A definitive prevention for T1D is not yet available, but current efforts focus on the identification of at‐risk individuals and therapies delaying the onset of Stage 3 T1D [5, 6, 7, 8, 9]. In this review, we present up‐to‐date knowledge related to the prevention of T1D in youth, involving disease‐modifying therapies at different stages. A narrative literature review utilising the PubMed/MEDLINE database was performed using the keywords ‘screening’, ‘prevention’, ‘Disease modifying therap*’, and ‘Diabetes Mellitus, Type 1’ [Mesh] in youth aged 0–18 years.

## 2 Identification of At‐Risk Individuals With Screening Programs

Diabetic ketoacidosis (DKA) is an acute and life‐threatening condition occurring in 15%–70% of children at the T1D onset, associated with considerable morbidity, need for hospitalisation, and risk of mortality [10]. Awareness campaigns on the warning signs of hyperglycemia have proven only partially effective in reducing the DKA incidence [11]. T1D screening in the general population or in the First‐Degree Relatives (FDR) individuals has recently been shown to be successful in reducing the incidence of DKA at diagnosis by early identification of IAb+ individuals and ongoing monitoring for progression towards Stage 3 T1D. With early identification of presymptomatic T1D and regular follow‐up, the rate of DKA drops significantly [12, 13, 14]. Education of the individuals IAb+ and their families has to include how to detect signs and symptoms of Stage 3 T1D and how to do so in case they appear [15].

The risk and rate of developing Stage 3 T1D vary depending on IAb status and age at seroconversion [12], and in individuals with multiple IAb+, the 15‐year risk of progression to Stage 3 type 1 diabetes varies from 18% to 88% [16]. Moreover, the rate of progression varies greatly between individuals and requires longitudinal monitoring, and individuals may occasionally revert from Stage 2 to Stage 1 [17, 18, 19]. All this uncertainty can lead to chronic anxiety, and a psychological support should be integrated into follow‐up, whenever possible, delivered by providers with diabetes‐specific training [20, 21, 22]. Some factors have been associated with higher anxiety during screening programs for T1D: parents from an FDR family, parents of children developing IAb+, parents perceiving that the child had a high risk of T1D, and parents with a lower education level [22].

Algorithms for monitoring people with IAb+, considering modality and frequency, are reported in different consensus and depend on age, length of time since the first detection of IAb+, number of IAb+ detected, and presence or absence of symptoms of T1D [3, 23, 24, 25]. However, there are still some debated points for which we need more evidence, and are reported here:

### 2.1 Who Should Be Screened?

General population screening programs, as well as FDR ones, have been introduced across Europe and the United States [26, 27]. FDR of people with T1D have a 15‐fold higher risk of developing Stage 3 T1D compared to the general population; however, 90% of newly diagnosed Stage 3 T1D individuals do not have FDR with T1D. IAb positivity could range between 0.7% and 7.2% in the general population and 0.9%–23.4% in the high‐risk/FDR population, and local cost‐effectiveness analysis should be considered for each screening programme [28, 29]. In some studies, IAbs screening was performed in individuals with HLA predisposition or based on a polygenic risk score [30], whereas in a clinical context IAbs screening is the one recommended.

### 2.2 Optimal Timing of Screening

Most children develop IAbs+ at a young age, whereas some individuals seroconvert later in life. Screening with IAbs has different sensitivity at different ages, and screening programs can consider different landmark ages [31, 32, 33]. Screening once for the presence of multiple islet autoantibodies has the highest sensitivity at age 4 years; this detects 40% of all cases of T1D by age 15. Screening twice (e.g., at ages 2 and 6) results in a sensitivity of > 80% [34]. An important aspect to consider is that the reimbursement of the costs involved with the monitoring of these individuals may vary from country to country.

### 2.3 How to Follow Up IAbs+ Individuals

If the IAb positivity is confirmed on a second separate serum sample, staging of T1D is determined by fasting glucose, 2‐h OGTT glucose and HbA_1c_. After staging, a debated point that needs more evidence is about the follow‐up modality of IAbs+ individuals. There are multiple available tools for monitoring IAbs+ individuals, including self‐monitored blood glucose (SMBG), continuous glucose monitoring (CGM), a standard OGTT, random venous glucose, and HbA1c. Different combinations of these tools and at different timings, according to the number of IAbs+ and age of the individual, are indicated by consensus panels [3, 23, 24, 25].

All individuals in the early Stages of T1D should be given a glucometer to measure finger‐prick blood glucose levels if they become symptomatic. Regarding OGTT, in individuals with multiple IAbs+, its metrics surpass CGM data for T1D prediction [35], but the burden and the organisational costs should be considered, particularly in individuals less than 2 years. Evidence is emerging on the value of periodic CGM to early detect dysglycaemia and predict the risk of progression to Stage 3 of T1D [36, 37, 38, 39, 40]. Children with more than 10% of time greater than 140 mg/dL over a 2‐week period have a risk of 80% to progress to Stage 3 T1D in 1 year [38]. In longitudinal models, repeated CGM and HbA1c were nearly as effective as OGTT in predicting Stage 3 T1D [41].

### 2.4 Where Should Monitoring Take Place?

Since many children and adolescents are monitored in primary care, this is an appropriate setting for recruiting healthy youths and performing capillary IAb tests; the first positive test has to be confirmed with a second one within 3 months [3]. Monitoring of individuals with IAb positivity needs different settings with diverse healthcare resources. Primary‐care providers can monitor Stage 1 T1D, according to routine metabolic follow‐up (SMBG, HbA1c, IAb), in collaboration with a multidisciplinary diabetes team (for education, psychological support, standard OGTT, CGM). If an IAb+ individual meets the criteria for stage 2 T1D, a referral should be made to the diabetes team, as well as an immediate consultation is needed if the individual develops symptomatic hyperglycemia [3]. Surveillance frequency should depend on the risk of progression, and all families need to be counselled about the expected progression to Stage 3 T1D. This monitoring programme requires an important educational, logistic, and organisational effort, with partnership between primary and secondary/tertiary care, communication, and coordination of care; the structure of the local healthcare system and available resources also have to be considered.

### 2.5 Ethical Concerns

Include the potential psychological distress from positive results in a population‐wide screening. The advantage of knowing in advance the possibility that a child has T1D, reducing the frequency of DKA and possibly delaying the diagnosis with a drug, must be balanced with the anxiety that this information produces in families and costs [3]. Positive IAb screening results in children may be associated with parental stress, depressive symptoms, and diabetes‐specific anxiety [20, 21, 22].

Parental distress and anxiety have been reported to increase at the time of IAb+ diagnosis but return to baseline levels with appropriate education and monitoring [20, 21, 22]. Moreover, psychological stress is comparable to that observed in families of children diagnosed with Stage 3 T1D [27].

Emotional, cognitive, and behavioural functioning assessment is important in children‐adolescents IAb+ and their families, and appropriate information and psychological support have to be delivered [3, 27].

### 2.6 Cost‐Effectiveness

Screening in the general population and follow‐up to age 18 was estimated to be cost‐saving in case of reducing the DKA rate by 20% and lifetime HbA1c by 0.1% [42]. Screening costs include initial outreach and recruitment, sample acquisition, time per patient screened (min), time cost per patient for recruiting, consenting, questionnaires, sample analysis, communicating results, metabolic staging, and education. These costs vary according to the types of IAb assays used and the population screened (general population vs. high‐risk individuals/FDR) [42, 43, 44, 45]. It should be noted that these considerations did not include the costs of any therapy to delay Stage 3 T1D. The only drug approved to delay the progression of Stage 3 T1D is Tzield (Teplizumab, Provention Bio), and a single, 14‐day course of treatment has a list price of approximately $193,900 in the United States [46]; only if the price of teplizumab is below $48,900, treating at‐risk individuals aged 8–49 years is cost‐effective [46].

### 2.7 Primary Prevention (Pre‐Stage 1)

Primary prevention should begin during pregnancy and focus on modifying risk factors that predispose individuals to the development of autoimmunity against pancreatic β‐cells. Several trials have targeted FDR with high genetic risk associated with HLA Class II genes. Different non‐pharmacological interventions have been proposed, including breastfeeding, hydrolysed infant formulae, gluten‐free diet, supplementation with omega‐3 fatty acids, calcitriol, or probiotics. However, these approaches have shown weak or no protective effects on the risk of T1D [47, 48, 49, 50, 51, 52]. Overall, current evidence does not support specific dietary recommendations for infants at increased risk of T1D.

Studies on oral insulin antigen administration confirmed its safety in infancy but unfortunately failed to demonstrate a protective immune response [53, 54].

### 2.8 Secondary Prevention: Therapies to Delay Stage 3 T1D

Identification of IAbs+ individuals allows participation in therapeutic options addressing the underlying pathogenesis of T1D, with the potential to preserve endogenous insulin secretion and delay or reduce the need for insulin replacement. We are now entering a new era where disease‐modifying therapies are becoming available in pre‐symptomatic T1D to halt the progression of the disease. In 2022, the US Food and Drug Administration (FDA) approved the first treatment to delay T1D: Teplizumab is clinically available and provides clear potential to intervene early and modify the course of T1D. Other immunomodulatory and/or immunosuppressive agents have been studied alone or in combinations to control or delay the progression of beta‐cell destruction, and a summary of the recent evidence from primary to tertiary prevention is here reported.

Secondary prevention is implemented during Stage 1 and Stage 2 T1D, when the autoimmune process has already been established. The aim was to delay progression to Stage 3 T1D in people with IAbs+.

**FIGURE 1:**
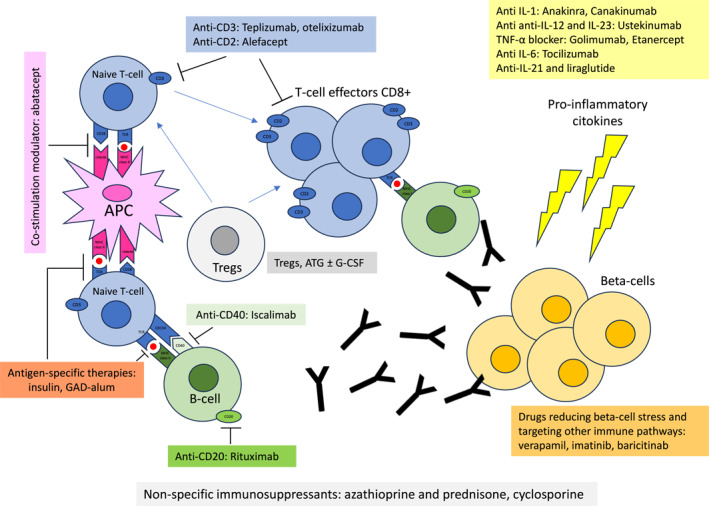
Summary of secondary and tertiary prevention strategies.

Secondary prevention has mainly focused on antigen‐specific therapies or monoclonal antibodies targeting specific immune cells (Figure 1, Table S1).

– *Oral or parenteral insulin* was evaluated in The Diabetes Prevention Trial, Type 1 (DPT‐1) [55, 56], and in the T1D TrialNet Oral Insulin Study [57], showing no delay in progression to Stage 3 T1D. Similarly, other clinical trials investigating intranasal insulin reached the same conclusion [58, 59]. More recently, a post‐hoc analysis of the TrialNet Oral Insulin Study reported that in a subcohort of stage 1–2 T1D patients carrying human leucocyte antigen (HLA) DR4‐DQ8 haplotype, oral insulin delayed progression to stage 3 onset (HR 0.59; *p* = 0.027), particularly in participants with high IA‐2A levels (HR 0.50; *p* = 0.028) [60].
– *Monoclonal antibodies targeting specific immune cells* have also been explored to delay the decline in beta‐cell function.

*Teplizumab* is currently the most advanced immunological therapy for the prevention of stage 3 T1D. It is a humanised monoclonal antibody of the immunoglobulin G1 type, which binds with high affinity to the ε chain of CD3. Teplizumab blocks the binding of CD3 to the T cell receptor and subsequently prevents the activation of T cells, in particular CD8+ T lymphocytes, which are involved in the destruction of beta‐cells [61]. Furthermore, anti‐CD3 monoclonal Abs increase CD4+ Tregs, thus promoting self‐tolerance [62]. Induction of genes associated with T‐cell exhaustion, immune regulation, and impaired T‐cell expansion—such as reduced expression of IL‐7 receptors—has been observed even 18 months after a single course of treatment in individuals at risk for T1D [63]. In November 2022, Teplizumab received FDA approval in the United States to delay the onset of T1D from stage 2 to stage 3 in adults and paediatric patients aged 8 years and older with stage 2 T1D [64]. The Phase 2 TN‐10 study (registered as clinical trial NCT01030861), published in 2019 in the New England Journal of Medicine, was a randomised, placebo‐controlled, double‐blind trial, and eligible participants were non‐diabetic relatives of patients with T1D at high risk of developing clinical diabetes [65]. The extension study demonstrated that 2‐week treatment with Teplizumab delayed by 2.7 years on average the onset of Stage 3 T1D in high‐risk participants [66]. In this population, the response to teplizumab was greater among patients with lower baseline C‐peptide levels, suggesting that teplizumab may be more beneficial for individuals with a more significant loss of beta‐cell function and, consequently, dysglycaemia [65]. Teplizumab is administered daily for 14 days by intravenous (iv) infusion (over a minimum of 30 min). Cytokine release is expected during the initial days of teplizumab infusion, consistent with CD3 agonism. Cytokine release syndrome (CRS), typically presenting with rash, headache, nausea, vomiting, and rigours, has been reported in 0.8% of patients across five clinical trials (*n* = 791). No grade 4 or fatal CRS events were observed. Other adverse effects were generally mild and self‐limited, including lymphopenia, leucopenia, elevated alanine aminotransferase, and gastrointestinal symptoms [67].

*Abatacept* (a CTL‐associated‐antigen 4 immunoglobulin binding to CD80/86) is a drug that blocks co‐stimulation, meaning it interferes with the interaction between antigen‐presenting cells (APCs) and T cells, specifically by targeting the CD80/CD86 molecules on APCs and preventing them from activating CD4+ T cells [68]. Abatacept, administered monthly for 2 years, has been shown to delay the reduction of C‐peptide in individuals with stage 3 T1D [69]. However, in a cohort with autoimmunity but without dysglycaemia, the decrease in beta‐cell function with abatacept was parallel to that with placebo, and it did not prevent the progression to Stage 3 T1D [70].

*Viral vaccinations*: early childhood infections and infections shortly before the onset of autoimmunity may promote autoreactivity against islet cells, especially in genetically susceptible children. Vaccination against viruses responsible for the increased risk of autoimmunity, therefore, represents a potential strategy for the prevention of T1D. Recent evidence has suggested a protective effect of rotavirus vaccination [71].

### 2.9 Tertiary Prevention: Therapies to Preserve Beta‐Cell Function at Onset

Tertiary prevention has the main goal of preserving residual beta‐cells in individuals with T1D onset, reducing exogenous insulin requirement, and delaying the onset of complications. The main tertiary interventions can be categorised into four groups: non‐specific immunosuppressants, monoclonal antibodies targeting specific immune cells, agents targeting specific cytokines, antigen‐specific therapies, and other immunomodulant drugs (Figure 1, Table 1).

**TABLE 1:** Overview of the main tertiary prevention interventions.

Class	Drug/intervention	Status	Main results
Non‐specific immunosuppressants	Azathioprine + prednisone	—	Temporary remission; frequent adverse effects
	Cyclosporine A	—	No effect; frequent adverse effects
Monoclonal antibodies	Teplizumab (anti‐CD3)	Phase III completed	Preserved C‐peptide, reduced insulin dose and HbA1c at 2 years
	Otelixizumab (anti‐CD3)	Phase III completed	Preserved C‐peptide with less adverse effect at a dosage of 9 mg
	Rituximab (anti‐CD20)	Phase II completed	Preserved C‐peptide, lower HbA1c, and lower insulin requirement over 2 years
	Abatacept (CTLA‐4‐Ig)	Phase II completed	Slowed decline in C‐peptide over 2 years
	Alefacept (anti‐CD2)	Phase II completed	Improved C‐peptide AUC at 2 years
	Iscalimab (anti‐CD40)	Phase II ongoing	Pending results
	Frexalimab (anti‐CD40)	Phase II ongoing	Recruiting
Agents targeting specific cytokines	Ustekinumab (anti‐IL‐12/23)	Phase II completed	Preserved C‐peptide, no significant effect on HbA1c at 1 year
	Etanercept (TNF‐α blocker)	Phase I/II completed	Reduced HbA1c and increased endogenous insulin production at 24 weeks
	Golimumab (TNF‐α blocker)	Phase II completed	Better endogenous insulin production and less exogenous insulin use than placebo at 52 weeks
	Anakinra and Canakinumab (IL‐1 blockers)	Phase II completed	Safe but not effective
	Tocilizumab (IL‐6 blocker)	Phase II completed	Slow loss of residual beta cell function at 52 weeks
	Ladarixin (inhibitor of IL‐8‐R)	Phase II completed	No effect
Antigen‐specific therapy	NBI‐6024 (soluble altered peptide ligand)	Phase I completed	No effect
	GAD‐alum (± vitamin D)	Phase I completed	Subcutaneous trials unsuccessful; other approach (intra lymph nodal injection) more promising
	GABA (± GAD‐alum)	Phase I/II completed	No effect
Other immunomodulant drugs	Ex vivo expanded Tregs	Phase I completed	Sustained C‐peptide response in combination with rituximab for up to 2 years
	ATG (± G‐CSF)	Phase II completed	Preserved stimulated C‐peptide 1 year after ATG alone therapy; no effect of combined ATG ± G‐CSF
	Liraglutide	Phase III completed	Better endogenous insulin production and less exogenous insulin use than before treatment
	Anti‐IL‐21 + liraglutide	Phase II completed	Improved insulin secretion; benefits faded after treatment discontinuation
	Verapamil (calcium channel blocker)	Phase II completed	Preserved C‐peptide levels at 52 weeks
	Imatinib (multi‐tyrosine kinase inhibitor)	Phase II completed	Preserved 12‐month C‐peptide AUC
	Baricitinib (JAK inhibitor)	Phase II completed	Increased meal‐stimulated mean C‐peptide level at 48 weeks
	Abrocitinib, ritlecitinib (JAK inhibitors)	Phase II ongoing	Recruiting

### 2.10 Non‐Specific Immunosuppressants (Agents Causing Generalised Immunosuppression)

*Azathioprine and prednisone* induced temporary remission in T1D patients within 2 weeks of beginning insulin, but these effects were short‐lived, and most patients experienced adverse effects [72].

*Cyclosporine* treatment had no effect on beta‐cell function in people with recent‐onset T1D during the first 12 months of treatment [73]. Following discontinuation of the cyclosporine A (CsA) and after a mean of 13.8 months, the mean insulin dose more than doubled (+111%) compared to a modest increase in the former placebo patients (+21%). Similarly, the significantly higher mean basal and stimulated C‐peptide values of the CsA patients fell within half a year to the level in the placebo group. Glycaemic control was transiently worse in the former CsA patients [74]. The lack of long‐term benefit, coupled with the then‐emerging recognition of cyclosporine side effects (particularly renal disease), led to the virtual abandonment of this therapy in T1D.

### 2.11 Monoclonal Antibodies Targeting Specific Immune Cells

– *Teplizumab* (anti‐CD3) in individuals recently diagnosed with T1D showed a reduced decline in C‐peptide and persistent immunological responses up to 7 years after diagnosis of T1D in responder individuals. Insulin use and HbA1c levels were also significantly lower in the drug‐treated responders than in the drug‐treated non‐responders or control participants during the first 2 years [75].

Signs of T cell exhaustion, such as reduced expression of the IL‐7 receptor, have been associated with clinical responses among drug‐treated participants in analyses during the treatment period [61].

– *Otelixizumab* (anti‐CD3) is another humanised anti‐CD3 monoclonal antibody with a similar reduction in T‐cell activation in comparison to teplizumab. In a randomised, placebo‐controlled Phase 2 study, it was IV administered over 6 days at three different dosages (9, 18, 27 mg) to individuals with new‐onset T1D. At 6, 12, and 18 months of follow‐up, subjects in the 9 mg treatment group had partially preserved beta‐cell function with lower insulin requirements than the control group. No significant differences in HbA1c were observed. No beta‐cell function preservation was observed at otelixizumab 18 and 27 mg. The frequency and severity of adverse effects were dose‐dependent [76].
– *Rituximab* (anti‐CD20) is a monoclonal antibody specific for the B‐cell surface protein CD20, which is required for B‐cell activation and proliferation. It showed to induce significant B‐cell depletion, but the effect on beta‐cell IAb titres was minimal, suggesting a beneficial but temporary effect of B‐cell depletion due to altered cross‐talk between B cells and T cells [77]. Rituximab showed partial preservation of beta‐cell function over 24 months, with a slower decline in C‐peptide levels, lower HbA1c, and lower insulin requirement [78, 79]. Adverse events were substantially mild. No subject required treatment for hypogammaglobulinaemia [79].
– *Abatacept* (a CTL‐associated‐antigen 4 immunoglobulin binding to CD80/86), iv administered monthly for 2 years in individuals with stage 3 T1D preserved beta‐cell function. MMTT‐stimulated C‐peptide AUC was 59% higher in the treatment group than in the placebo group at 24 months. In addition, treated subjects maintained better HbA1c and had more insulin secretion 3 years after diagnosis than the placebo‐treated subjects [69].
– *Alefacept* (anti‐CD2) causes a selective reduction in circulating effector and memory T cells. Indeed, CD2 is expressed on all T cells but is most highly expressed by memory and effector T cells and is expressed at lower levels on naïve and regulatory T cells (Tregs) [80]. In a clinical trial, two 12‐week courses of intramuscular (IM) alefacept induced a significant response in 30% of treated individuals. They preserved endogenous insulin production as shown by maintained C‐peptide secretion, reduced insulin use and hypoglycemic events compared with placebo at 24 months [81].
– *Iscalimab* (anti‐CD40) is a fully human anti‐CD40 monoclonal antibody that targets the CD40‐CD154 co‐stimulatory pathway. This pathway leads to the T‐cell‐dependent humoural immune response and is essential for priming and activating CD4+ autoreactive T cells and CD8+ cytolytic T cells [82], resulting in attenuation of the B‐cell activation signal without depleting peripheral blood B cells. An ongoing Phase 2, multicenter, double‐blind, randomised, placebo‐controlled study will evaluate the safety, tolerability, pharmacokinetics, and efficacy of iscalimab in preserving residual pancreatic beta‐cell function in new‐onset T1D in paediatric and young adult subjects (NCT04129528). The trial is actively ongoing.
– Frexalimab is the first second‐generation antibody targeting CD40L to be investigated in T1D. Clinical trials are currently recruiting adults and adolescents (NCT06111586).

### 2.12 Agents Targeting Specific Cytokines

– *Ustekinumab* (anti‐IL‐12 and IL‐23) in adolescents with recent‐onset T1D showed that beta‐cell function was 49% higher in the intervention group compared with the placebo arm after 12 months. However, the reduction in beta‐cell destruction did not translate into a significant effect on other metabolic parameters, such as HbA1c, during the timeframe of the study [83].
– *Etanercept* (recombinant TNF‐α receptor‐IgG fusion protein) is an anti‐TNF‐α agent playing an important role as an intermediate molecule in several autoimmune diseases [84]. In children with newly diagnosed T1D (aged 7.8–18.2 years) led to a reduction in HbA1c and an increase in endogenous insulin production was assessed by C‐peptide levels [85].
– *Golimumab* (another anti‐TNF‐α agent), approved for the treatment of several autoimmune diseases, was tested for T1D in a phase II, multicenter, placebo‐controlled, double‐blind, parallel‐group study (T1GER). Eighty‐four participants (age range, 6–21 years) with newly diagnosed T1DM received either golimumab or placebo subcutaneously for 52 weeks. At week 52, the mean 4‐h C‐peptide AUC differed significantly between the golimumab and placebo groups (0.64 pmol/mL vs. 0.43 pmol/mL, *p* < 0.001). In addition, at week 52, the total daily insulin use was lower in the golimumab group than in the placebo group [86].
– *Anakinra and canakinumab* (anti‐IL‐1) failed to show significant C‐peptide preservation in Phase 2 studies in Stage 3 T1D [87].
– *Tocilizumab* (anti‐IL‐6) reduced T cell IL‐6 receptor signalling but failed to show a delay in the loss of residual beta‐cell functioning in newly diagnosed T1D [88].
– Ladarixin (an allosteric inhibitor of the IL‐8 receptors CXCR1/CXCR2) has been tested in the short term (400 mg twice daily for three cycles of 14 days on/14 days off) in adults, with no appreciable effect on preserving residual beta‐cell function [89]. A clinical trial including an estimated 15%–20% of adolescents is active but not currently recruiting (NCT04628481).

### 2.13 Antigen‐Specific Therapies (Immunologic Vaccination)

Antigen‐specific therapies, such as insulin and glutamic acid decarboxylase (GAD), aim to induce antigen‐specific CD4^+^CD25^high^FOXP3^+^ regulatory T cells (Tregs) and promote the secretion of tolerogenic cytokines, thereby reducing islet‐specific cytotoxic lymphocyte responses.

The insulin B (9–23) peptide has been identified as an important T‐cell antigen in T1D. NBI‐6024 is a *soluble altered peptide ligand* (APL) with the potential to block or modify this response. Nevertheless, subcutaneous injections of NBI‐6024, tested in 188 patients with recently diagnosed T1D, did not improve or preserve beta‐cell function [90].

GAD is one of the major autoantigens involved in the autoimmune process underlying T1D. GAD‐alum is Recombinant human GAD65 (rhGAD65) and is used as an antigen‐specific immune modulator. Subcutaneous injections of *GAD‐alum* did not significantly reduce the loss of stimulated C‐peptide or improve clinical outcomes over a 15‐month period [91]. Other approaches, such as injection into lymph nodes, seem to be more promising, particularly when combined with vitamin D supplementation [92, 93] and in patients carrying the HLA DR3‐DQ2 haplotype [93].

Gamma‐aminobutyric acid (GABA) is synthesised by GAD in pancreatic beta‐cells. GABA alone or in combination with GAD‐alum did not preserve beta‐cell function [94].

### 2.14 Other Immunomodulant Drugs

– *Autologous Polyclonal CD4+CD25+CD127lo/‐FOXP3+ Regulatory T‐cells (Tregs)* are a subset of CD4+ T cells that play an essential role in the induction and maintenance of peripheral tolerance and are essential for preventing excessive immune responses. In clinical trials, Tregs did not prevent a decline in residual beta‐cell function over 1 year compared to placebo in single dose [95]. However, over 2 years, combined therapy with Tregs and rituximab was consistently superior to monotherapy in delaying T1D progression in terms of C‐peptide levels and the maintenance of remission [96].
– *Anti‐thymocyte globulin (ATG) or a combination of ATG and pegylated G‐CSF* can help in the recovery of Tregs. ATG is a pasteurised solution consisting primarily of rabbit immunoglobulin G (IgG)‐derived polyclonal Abs directed against multiple T‐cell antigens [97]. At 12 months, the mean AUC C‐peptide was significantly higher in subjects treated with ATG (0.646 nmol/L) versus placebo (0.406 nmol/L) (*P* = 0.0003) [98]. On the contrary, combined ATG and G‐CSF treatment did not preserve stimulated C‐peptide in new‐onset patients with T1D within 24 months [98, 99], and after 5 years [100].
– *Liraglutide*, a glucagon‐like peptide‐1 receptor agonist, represents an important add‐on therapy for reducing insulin requirements, promoting weight loss, and achieving modest improvements in HbA1c levels in adults with T1D. The *combination of anti‐IL‐21 and liraglutide* has been shown to stabilise and preserve beta‐cell survival and function at week 54 in adult patients; however, the effects diminished after treatment cessation [101]. A trial testing liraglutide in five adolescents with T1D of more than 1‐year duration has recently been completed. Preliminary results demonstrated a reduction in total daily insulin dose in treated patients compared with pre‐liraglutide treatment (NCT02516657).
– *Verapamil* (calcium channel blocker) improves beta‐cell stress and survival via targeting of thioredoxin‐interacting protein overexpression, which has been shown to induce pancreatic beta‐cell apoptosis and glucotoxicity‐induced beta‐cell death. In children and adolescents with newly diagnosed T1D, verapamil partially preserved stimulated C‐peptide secretion at 52 weeks compared with placebo [102]. In adults, verapamil treatment versus placebo was associated with improved mixed‐meal–stimulated C‐peptide AUC at 3 and 12 months, smaller increases in insulin requirements, and better on‐target glycaemic control [103].
– *Imatinib* (Gleevec) is a multi‐tyrosine kinase inhibitor approved for the treatment of some types of leukaemias and solid tumours. Although its mechanism remains unclear, imatinib may act on both immunological and metabolic pathways involved in the development of T1D, preserving beta‐cells by counteracting elevated endoplasmic reticulum stress [104]. In adults with T1D, imatinib preserved 12‐month C‐peptide AUC out to 24 months [105]. A single case of imatinib use in an adolescent with T1D has been reported: the patient presented with diabetic ketoacidosis (DKA) and a concomitant myeloid neoplasm. Following initiation of exogenous insulin and imatinib, he experienced a 1.7‐point reduction in HbA1c and a 71% reduction in insulin requirements, with sustained partial diabetes remission [106].
– *Baricitinib* **(**JAK inhibitor) impairs cytokine‐induced major histocompatibility complex (MHC) class I expression in cultured islets and islet cells, impairs CD8+ T‐cell activation, and blocks the formation of immune synapses between beta‐cells and CD8+ T cells [107]. Daily treatment over 48 weeks was associated with an increased meal‐stimulated mean C‐peptide level [107]. Other clinical trials investigating JAK inhibitors, such as abrocitinib and ritlecitinib, are currently being conducted (NCT05743244).

## 3 Conclusion

T1D is a chronic autoimmune disease, and identification of individuals in the presymptomatic stages could slow or halt the progression to the symptomatic Stage 3. General population and FDR screening programs allow for the identification of individuals at Stage 1, Stage 2, or presymptomatic Stage 3 T1D who need to be monitored to avoid DKA, receive education, and psychological support.

Are we ready for national population‐wide screening programs? As monitoring programs of individuals IAb+ are available from expert consensus and regional screening programs, the main challenges involve coordination between different settings of care and locally available resources to support the costs of recruiting, sample analysis, metabolic staging, education, and psychological support.

At the same time, screening programs offer the possibility to enrol participants in clinical trials testing new therapies that can change the natural history, delaying the progression to overt T1D. Are we ready with the prevention of Stage 3 T1D? Although numerous clinical trials have been conducted or are still ongoing using disease‐modifying therapies, only Teplizumab has been approved by the FDA as the first drug shown to delay the onset of Stage 3 T1D. Several intervention trials in individuals with Stage 3 T1D have led to a temporary improvement in beta‐cell function, but this benefit has consistently been short‐lived. Identifying biomarkers of responsiveness is an important objective for understanding the mechanisms of the treatment and the disease, maximising efficacy, and avoiding treatment of those who are not likely to respond to Teplizumab. To date, no specific pre‐treatment biomarker predicts teplizumab response; however, potential indicators include baseline differences in T cell subsets [108, 109].

Although our understanding of the rate and mechanisms underlying progression across the different stages of T1D remains incomplete, including the role of environmental triggers such as viral infections, microbiome alterations, and dietary factors, the recent success of Teplizumab represents a major breakthrough: we have the opportunity to better understand, in individuals treated with this drug, the patient's disease progression and potential autoimmune triggers. Teplizumab has demonstrated the ability to delay the onset of stage 3 T1D in individuals at high risk, offering a crucial window of time for intervention. These results suggest that immune intervention during the presymptomatic stages of disease is not only possible but also clinically meaningful. As such, we are entering a new era in T1D management, one that moves beyond reactive treatment at diagnosis towards proactive, stage‐specific prevention strategies. Expanding beyond traditional monotherapies is essential in the future, investigating combinations of disease modifying drugs, and the development of personalised treatment strategies tailored to the unique characteristics of each individual with presymptomatic T1D.

## Author Contributions

**Evelina Maines:** validation, formal analysis, writing – original draft. **Roberto Franceschi:** conceptualization, methodology, formal analysis, writing – original draft, writing – review and editing. **Francesca di Candia:** validation, writing – original draft. **Enza Mozzillo:** conceptualization, methodology, formal analysis, writing – original draft, writing – review and editing. All authors have read and agreed to the published version of the manuscript.

## Funding

The authors received no specific funding for this work.

## Conflicts of Interest

The authors declare no conflicts of interest.

## Peer Review

The peer review history for this article is available at https://www.webofscience.com/api/gateway/wos/peer-review/10.1002/dmrr.70101.

## Supporting information


**Table S1:** Summary of evidence of the main secondary and tertiary prevention strategies for children and adolescents.

## Data Availability

The data that support the findings of this study are available from the corresponding author upon reasonable request.
