# Supplementary material for: Are We Ready With Prevention for Type 1 Diabetes?
Source: Diabetes Metab Res Rev. 2025 Oct 25;41(7):e70101. doi: 10.1002/dmrr.70101 (PMC12553304; doi:10.1002/dmrr.70101)
Supplement: Supplementary file 1 — Table S1: Summary of evidence of the main secondary and tertiary prevention strategies for children and adolescents. [file DMRR-41-e70101-s001.docx]

**Table S1:** summary of evidence of the main secondary and tertiary prevention strategies for children and adolescents

| **Reference** | **Study design** | **Sample, age and comparator** | **Intervention** | **Main results** | **Study limitations, risk of bias** |
| --- | --- | --- | --- | --- | --- |
| **Secondary prevention** | | | | | |
| Diabetes Prevention Trial-Type 1 Diabetes Study Group, 2002  [55] | RCT (DPT-1 trial) | n. 339 first-degree and second-degree relatives IAb+ of patients with T1D (median age 11.2 y)  n. 169 intervention group  n. 170 observation group | Low-dose s.c. ultralente insulin, twice daily for a total dose of 0.25 U/kg/d, plus annual four-day continuous iv infusions of insulin  Median follow-up: 3.7 y | 40.8% of patients in the intervention group vs 41.2% in the observation group progressed to stage 3 T1D. The annualized rate of progression was 15.1% in the intervention group and 14.6% in the observation group. The cumulative incidence of stage 3 T1D was similar in the two groups (HR in the intervention group as compared with the observation group, 0.96; P = 0.80) |  |
| Skyler JS et al, 2005  [56] | RCT (DPT-1 trial) | n. 372 first-degree and second-degree relatives IAb+ of patients with T1D (median age 10.2 y)  n. 186 oral insulin  n. 186 placebo | Oral insulin (7.5 mg/d) or placebo  Median follow-up: 4.3 y | 23.7% of patients in the oral insulin group vs 28.5% in the placebo group progressed to stage 3 T1D. Annualized rate of diabetes was similar in both groups: 6.4% with oral insulin and 8.2% with placebo (HR 0.764, P = 0.189) |  |
| Krischer JP et al, 2017  [57] | RCT (TrialNet Oral Insulin trial) | n. 560 first-degree and second-degree relatives IAb+ of patients with T1D (median age, 8.2 y)  n. 283 oral insulin  n. 277 placebo | Oral insulin (7.5 mg/d) or placebo  Median follow-up: 2.7 y | Stage 3 T1D was diagnosed in 58 participants (28.5%) in the oral insulin group and 62 (33%) in the placebo group. Time to diabetes was not significantly different between the 2 groups (HR, 0.87; P = 0.21) |  |
| Harrison LC et al, 2004  [58] | RCT cross-over | n. 38 first-degree relatives IAb+ of patients with T1D (16 females and 22 males, mean age 10.8 y) recruited from the Melbourne Pre-Diabetes Family Study  4 had one IAb+; 22 two IAbs+ and 12 three IAbs+ | Intranasal insulin (I) (1.6 mg) or a carrier solution (P, placebo), daily for 10 days and then 2 days a week for 6 months, before crossover  Randomization to 2 arms (two 6-month treatment periods): in the I/P arm, period 1 = I and period 2 = P; in the P/I arm, period 1 = P and period 2 = I  n. 20 I/P arm  n. 18 P/I arm | Stage 3 T1D developed in 12 participants (6 randomized to each arm) after a median of 1.1 year (range 0.3-3.6 y) | Small sample size  Technical difficulties in intranasal insulin self-administration  Possible genetic heterogeneity in the response to insulin as an autoantigen |
| [Näntö-Salonen](https://pubmed.ncbi.nlm.nih.gov/?sort=pubdate&term=N%C3%A4nt%C3%B6-Salonen+K&cauthor_id=18814906) K et al, 2008  [59] | RCT | n. 224 infants and 40 siblings with two IAbs+ and HLA-DQB1 susceptibility alleles  n. 137 insulin  n. 127 placebo | Short-acting human insulin (1 U/kg) once a day intranasally or placebo  Median follow up: 1.8 y (range 0-9.7 y) | Administration of nasal insulin, started soon after detection of IAbs+, did not prevent or delay T1D. n. 56 in the insulin group, and n. 53 in the placebo group developed stage 3 T1D (HR 0.98; P = 0.91) |  |
| Zhao LP et al, 2024  [60] | Post hoc TrialNet oral insulin trial analysis | n. 560 first-degree and second-degree relatives IAb+ of patients with T1D (median age, 8.2 y)  n. 283 oral insulin  n. 277 placebo | Oral insulin (7.5 mg/d) or placebo  Median follow-up: 2.7 y | Oral insulin reduced the frequency of T1D onset among participants with elevated IA-2A levels (HR 0.62; P = 0.012) but had no preventive effect among those with low IA-2A levels (HR 1.03; P = 0.91). High IA-2A levels were positively associated with the HLA DR4-DQ8 haplotype (OR 1.63; P = 6.37 × 10−6) and negatively associated with the HLA DR7–containing DRB1*07:01-DRB4*01:01-DQA1*02:01-DQB1*02:02 extended haplotype (OR 0.49; P = 0.037). Among DR4-DQ8 carriers, oral insulin delayed the progression toward stage 3 T1D onset (HR 0.59; P = 0.027), especially if participants also had high IA-2A level (HR 0.50; P = 0.028). | Post-hoc analysis |
| Herold KC et al, 2019  [65] | Phase 2, placebo-controlled, double-blind, RCT (TrialNet Anti-CD3 Prevention - TN10) | n. 76 FDR of patients with T1D with two or more IAbs+ AND dysglycaemia during an OGTT within 52 days before enrollment.  n. 44 teplizumab group  n. 32 placebo group  72% 8-18 y of age  Median age: 13 y (range 8-49 y) | Single 14-day course of teplizumab or placebo  Median follow up: 51 months | 43% of patients in the Teplizumab group vs 72% in the placebo group progressed to stage 3 T1D. The median time to progression was 48.4 months in the teplizumab group vs 24.4 months in the placebo group (HR 0.41, P = 0.006).  The most pronounced effect of teplizumab was observed within the first year: only 7% of the patients in the Teplizumab group were diagnosed with disease vs 44% of those in the placebo group.  The annualized rates of diagnosis of T1D were 14.9% per year in the teplizumab group and 35.9% per year in the placebo group.  The response to teplizumab was greater among participants whose C-peptide responses to the OGTT at baseline were below the median (1.75 nmol per liter) than among those whose responses were above the median (HR, 0.19; 95% CI, 0.08 to 0.47) | Small sample size  The drug was given for only one course  Mixed sample  (adults and children) |
| Sims et al, 2021  [66] | Extension of TrialNet Anti-CD3 Prevention - TN10 trial | n. 76 FDR with stage 2 T1D  Median age: 13 y (range 8-49 y) | Single 14-day course of iv teplizumab or placebo  Median follow up: 76.9 months | 50% of patients in Teplizumab group vs 78% in the placebo group progressed to stage 3 T1D  The median time to progression was 59.6 months in the teplizumab group vs 27.1 months (Δ 2.7 years) in the placebo group (HR = 0.457, P = 0.01). | Small sample size  The drug was given for only one course  Mixed sample  (adults and children) |
| Russel WE et al, 2023  [70] | Phase 2, placebo-controlled, double-blind RCT | n. 212 relatives IAb+ (n.134 <18 y, n. 78 ≥18 y) of patients with T1D, from the TrialNet Pathway to Prevention screening program (patients with Stage 1 T1D)  n. 101 abatacept  n. 111 placebo | 14 iv infusions of abatacept (10 mg/kg up to a maximum of 1000 mg) or placebo at 0, 2, and 4 weeks after randomization and then every 28 ± 7 days for 12 months  Median follow up: 47.6 months | The study did not demonstrate a statistically significant delay in progression to stage 2 or 3 T1D.  n. 81 participants (35 abatacept and 46 placebo) developed abnormal glucose tolerance or stage 3 T1D (HR 0.702; P = 0.11) | Mixed sample (adults and children) |
| **Tertiary prevention** | | | | | |
| **Non-specific immunosuppressants** | | | | | |
| [Silverstein](https://pubmed.ncbi.nlm.nih.gov/?sort=pubdate&term=Silverstein+J&cauthor_id=3045545) J et al, 1988  [72] | RCT | n. 46 patients (mean age 11.7 y; range 4.5-32.8 y) with newly diagnosed T1D (within two weeks of beginning insulin)  n. 20 immunosuppression  n. 26 no immunosuppression | Corticosteroids (prednisone) for 10 weeks plus daily azathioprine for one year, or no immunosuppression  Follow up: 1 year | 50% of immunosuppressed patients completing the one-year trial had satisfactory metabolic outcomes (HbA1c less than 6.8%; stimulated peak C peptide greater than 0.5 nmol/L; insulin dose less than 0.4 U/Kg/d) as compared with only 15% of the controls. 3/20 immunosuppressed patients, but no controls, were insulin independent at one year.  The side effects of azathioprine included vomiting in one patient and mild hair loss in several others. Prednisone use resulted in a transient cushingoid appearance, weight gain, and hyperglycemia. | Small sample size  Mixed sample  (adults and children) |
| Skyler JS, et al, 1992  [73] | RCT (Miami group study) | n.23 subjects (mean 19.7 +/- 1.8 y; range 9-38 y) enrolled within 6 weeks of T1D diagnosis. | Cyclosporine (initial dosage 10 mg/kg/d), given as a single daily dose, adjusted on the basis of side effects and trough cyclosporine levels  Follow up: 1 year | Glycemic control and insulin dosage were similar in both cyclosporine and placebo groups. The frequency of freedom from insulin usage also was similar in both groups. | Small sample size  Mixed sample  (adults and children) |
| Martin S et al, 1991  [74] | RCT (Extension of the Canadian/European RCT) | n. 188 recent onset T1D patients (age 9-35 y, duration of classic symptoms before onset < 14 weeks) | Cyclosporine A (CsA) vs placebo  The initial CsA dose was 10 mg/kg divided in 12 h intervals, subsequently the dose was modified to maintain 12 h trough concentrations in serum of 100-200 ng/mL or in whole blood of 400-800 ng/mL | CsA administration was discontinued at a mean of 13.8 +7.4 months (SD). Within 6 months the mean insulin dose more than doubled (+ 111%) compared to a modest increase in the former placebo patients (+ 21%). Similarly, the significantly higher mean basal and stimulated C-peptide values of the CsA patients fell within half a year to the level of the former placebo patients. Glycaemic control was transiently worse in the former CsA patients. | Mixed sample  (adults and children) |
| **Monoclonal antibodies** | | | | | |
| Perdigoto AL et al, 2019  [75] | Prospective observational study (part of AbATE trial). | n. 43 patients (age range 8-30 y), Iab+ (anti-GAD65, anti-ICA512 or ICA), and newly diagnosed with T1D (within 8 weeks of enrolment.  n. 31 teplizumab group)  n. 12 control group | Protocol of AbATE trial:14-day course of teplizumab administered iv (median cumulative dose 11.6 mg) at study entry. 40/52 individuals in the drug treatment group received a second dose of teplizumab (median cumulative dose 12.4 mg)  Randomization 2:1 ratio, drug:control  Drug-treatment response: absence of a change in C-peptide from baseline to 1 year (i.e. <7.5%)  Median follow-up: 7 y | The C-peptide responses to a MMTT were similar overall in the drug vs control group of participants at the follow-up visit but were significantly improved, with less loss of C-peptide, in drug-treated responders identified at 1 year.  Insulin use and HbA1c levels were significantly lower in the drug-treated responders than in the drug-treated non-responders or control participants at visits during the first 2 years but not at the follow-up visit. | Small sample size  Mixed sample  (adults and children) |
| Keymeulen B et al, 2021  [76] | Phase 2, placebo-controlled, single-blind RCT | n. 30 patients (age range 16–27 y) with recent onset T1D (32 days) + for at least one Abs; stimulated C-peptide peak level of ≥0.2 nmol/L after MMTT | iv otelixizumab  Randomization to daily iv infusions over 6 days:  n. 6 placebo  n. 9 otelixizumab 9 mg  n. 8 otelixizumab 18 mg  n. 7 otelixizumab 27 mg | Change from baseline MMTT C-peptide weighted mean AUC 0-120 min following otelixizumab 9 mg was above baseline for up to 18 months (difference from placebo 0.39 [95% CI 0.06, 0.72]; P = 0.023); no beta cell function preservation was observed at otelixizumab 18 and 27 mg.  The frequency and severity of adverse effects were dose dependent  EBV reactivation was dose dependent and transient. | Small sample size  Mixed sample  (adults and children) |
| Herold KC et al, 2011  [78] | Placebo-controlled  RCT | n. 78 completed the 1 year-MMTT. Based on the change in the C-peptide AUC from baseline to 6 months, each participant was designated as a C-peptide responder or non-responder.  n. 52 rituximab (responders n.30, age 19.1 ± 8.8 y; non responders n.21, age 19.8 ± 8.9 y)  n. 29 placebo (responders 11, age 19.6 ± 9.5 y; non responders n. 18, age 16.0 ± 6.8 y) | Randomization 2:1 ratio to rituximab or placebo  Follow-up: 12 months | Based on the observed change in C-peptide responses relative to the coefficient of variation (CV) of repeated measurements, 58% of the subjects in the rituximab treated group were responders.  T-cell proliferative responses to diabetes–associated antigens were present at baseline in 75% of anti-CD20- and 82% of placebo-treated subjects and were not different over time. However, in rituximab-treated subjects with significant C-peptide preservation at 6 months (58%), the proliferative responses to diabetes associated total (P = 0.032), islet-specific (P =0 .048), and neuronal auto-antigens (P = 0.005) increased over the 12-month observation period. This relationship was not seen in placebo treated patients. | Mixed sample (adults and children) |
| Pescovitz MD et al, 2014  [79] | Phase 2 RCT | n.87 patients with newly diagnosed T1D (age 8-40 y)  n.57 rituximab  n.30 placebo | Randomization 2:1 ratio to:  - one course of four iv infusions of rituximab (each infusion being 375 mg/m2)  - placebo  Follow-up: 24 months | The rituximab group had lower HbA1c (6.76 ± 1.24 versus 7.00 ± 1.30% at 12 months; P ≤ 0.0001) and insulin dose (0.39 ± 0.22 versus 0.48 ± 0.23 U/kg at 12 months; P ≤ 0.0001). Both values were lower in the rituximab group at 18 and 24 months but were no longer significantly different from the placebo group.  IgM levels fell from baseline in the rituximab group, an effect that persisted at 24 months (P < 0.0001). The IgG concentrations did not differ significantly between the two groups and remained similar to that at baseline | Mixed sample (adults and children) |
| Orban T et al, 2014  [69] | Phase 2 RCT | n. 112 T1D patients (age 6-45 y)  -Abs +,  -diagnosis of T1D <100 days,  -C-peptide stimulated levels of ≥0.2 pmol/mL during a MMTT  n. 77 abatacept  n. 35 placebo | Randomization in a 2:1 ratio to:  -Abatacept iv administered on days 1, 14, and 28, and then every 28 days, with the last administration on day 700 (month 24; 27 total doses) as a 30-min infusion (10 mg/kg; maximum 1,000 mg/dose)  -Placebo (normal saline solution infusion)  Follow-up: 30 months | C-peptide AUC means, adjusted for age and baseline C-peptide, at 36 months were 0.217 nmol/L (95% CI 0.168-0.268) and 0.141 nmol/L (95% CI 0.071-0.215) for abatacept and placebo groups, respectively (P = 0.046). The C-peptide decline from baseline remained parallel with an estimated 9.5 months' delay with abatacept. Moreover, HbA1c levels remained lower in the abatacept group than in the placebo group. The slightly lower (nonsignificant) mean total insulin dose among the abatacept group reported at 2 years was the same as the placebo group by 3 years.  Subjects who received abatacept had a significantly higher mean AUC of 28%, 30%, 38%, 59%, 48%, and 54% compared with placebo subjects at 6, 12, 18, 24, 30, and 36 months, respectively. | Mixed sample (adults and children) |
| Rigby MR et al, 2015  [81] | Phase 2 RCT | n. 49 T1D patients (age 12–35 y) (<100 days from diagnosis); + for at least one Abs; with a peak-stimulated C-peptide of >0.2 nmol/L during a MMTT.  n. 33 patients received alefacept  n. 16 placebo | Randomization in a 2:1 ratio to:  - Alefacept 15 mg im  - Placebo equivalent volume of saline im weekly for 12 weeks and, after a 12-week pause, 12 additional weekly doses of alefacept or placebo.  Participants underwent a 4-hour MMTT at screening, 52 weeks, and 104 weeks; a 2-hour MMTT at 24 and 78 weeks  Follow-up: 24 months | At 24 months the 4-hour and the 2-hour C-peptide AUCs were significantly greater in the treatment group than in the control group (P = 0.002 and 0.015, respectively).  Insulin requirements were lower (P = 0.002) and rates of major hypoglycemic events were about 50% reduced (P < 0.001) in the alefacept group compared with placebo at 24 months. | Small sample size  Mixed sample  (adults and children) |
| **Agents targeting specific cytokines** | | | | | |
| Tatovic D et al, 2024  [83] | Phase 2 RCT | n.62 adolescents (age 12-18 y) with recent-onset T1D  n. 41 Ustekinumab  n. 21 placebo | Ustekinumab iv (7 doses)  Follow-up: 12 months | Ustekinumab was associated with a difference of 49% higher C-peptide AUC in the treatment group at week 52 (P = 0.02)  The reduction in beta cell destruction did not translate into a significant effect on other metabolic parameters (HbA1c, time in range on CGM and IDAA1c) during the timeframe of the study | Small sample size |
| Mastandrea L et al, 2019  [85] | Double-blind, randomized, placebo-controlled | n.18 subjects (11 male and 7 females, aged 7.8-18.2 y) with new-onset T1D (≤4 weeks), GAD-65 and/or islet cell antibody positivity, A1c >6%, three insulin injections per day, white blood cell count 3,000–10,000, platelets >100,000, and normal liver and renal function. | Etanercept vs placebo  Etanercept: 0.4 mg/kg up to a maximum dose of 25 mg/dose s.c. twice weekly for 24 weeks  Follow-up: 24 weeks | A1c at week 24 was lower in the etanercept group (5.91 ± 0.5%) compared with that in the placebo group (6.98 ± 1.2%; P < 0.05) with a higher percent decrease from baseline than in the placebo group (etanercept 0.41 ± 0.1 vs. placebo 0.18 ± 0.21; P < 0.01). The percent change in C-peptide area under the curve from baseline to week 24 showed a 39% increase in the etanercept group and a 20% decrease in the placebo group (P < 0.05). From baseline to week 24 insulin dose decreased 18% in the etanercept group compared with a 23% increase in the placebo group (P < 0.05). Seventeen patients completed the study, and none withdrew because of adverse events. | Small sample size |
| Quattrin T et al, 2020  [86] | Phase 2, multicenter, placebo-controlled, double-blind, parallel-group RCT | n. 84 children and adolescents (age 6-21 y) with recent onset of T1D (within 100 days) with at least one IAbs+ and a peak of C-peptide level of at least 0.2 pmol/mL after MMTT | Randomization in a 2:1 ratio to:  - golimumab s.c. (if body weight <45 kg induction dose of 60 mg/m2 at weeks 0 and 2; if >=45 kg induction dose of 100 mg at weeks 0 and 2. Maintenance s.c. doses of 30 mg/m2 and 50 mg/m2 at week 4 and every 2 weeks through week 52)  - placebo  Follow-up: 52 weeks | A partial-remission response was observed in 43% of participants in the golimumab group and in 7% of those in the placebo group (difference, 36 percentage points; 95% CI, 22 to 55).  The mean change from baseline in the 4-hour C-peptide AUC was –0.13 pmol/mL (95% confidence interval [CI], –0.23 to –0.03) in the golimumab group and –0.49 pmol/mL (95% CI, –0.66 to –0.32) in the placebo group, which represents a mean percent decrease of 12% in the golimumab group and of 56% in the placebo group.  The mean number of hypoglycemic events did not differ between the trial groups.  At week 52, the total daily insulin use was lower in the golimumab group than in the placebo group (0.51 U/kg/d vs. 0.69 U/kg/d) | Small sample size |
| Moran A et al, 2013  [87] | Phase 2a  Placebo-controlled RCT | 2 studies:  TrialNet Canakinumab vs placebo  IDA trial Anakinra vs placebo  Canakinumab:  n. 69 patients (age 6-45 y) with onset of T1D diagnosed within 100 days; at least one IAbs+; and a peak C-peptide concentration of at least 0.2 nmol/L after a standardized MMTT21 days after diagnosis of T1D and within 37 days of randomization  (n. 47 Canakinumab; n. 22 placebo)  Anakinra:  n. 69 patients (age 18-35 y) with onset of T1D within 12 weeks; +GAD-65; a peak C peptide of at least 0.2 nM after a standardized MMTT  (n. 35 Anakinra; n. 35 placebo) | The TrialNet canakinumab study used 2:1 randomization:  monthly s.c. injections of 2 mg/kg (maximum 300 mg) canakinumab or an identically appearing placebo for 12 doses. The next dose was rescheduled in participants with signs of active infection within the previous 48 h.  Follow-up: 12 months  The AIDA trial used 1:1 randomization: recombinant human IL-1 receptor antagonist anakinra at the daily dose of 100 mg as a single, self-administered, s.c. injection every morning for 9 months or placebo  Follow-up: 9 months | The difference in C peptide AUC between the canakinumab and placebo groups at 12 months was 0.01 nmol/L (95% CI -0·11 to 0·14; P = 0.86), and between the anakinra and the placebo groups at 9 months was 0.02 nmol/L (-0·09 to 0·15; *P* = 0.71).  The number and severity of adverse events did not differ between groups in the canakinumab trial. In the anakinra trial, patients in the anakinra group had significantly higher grades of adverse events than the placebo group (P = 0.018). | Different inclusion criteria  Different protocols  AIDA trial no pediatric patients  Mixed sample  (adults and children) |
| Greenbaum CJ et al, 2021  [88] | Placebo-controlled, multicenter, double-blind RCT | n. 81 pediatric patients (age 6-17 y) within 100 days of T1D diagnosis with at least 1 diabetes-related IAb+, C-peptide ≥ 0.2 pmol/mL during an MMTT  n. 55 adults (age 18-45 y)  n. 54 pediatric patients: tocilizumab  n. 27 pediatric patients: placebo  n. 35 adults: tocilizumab  n. 20 adults: placebo | Randomization in a 2:1 ratio to:  - Tocilizumab iv, if at least 30 kg body weight at a dose of 8 mg/kg to a maximum of 800 mg; if less than 30 kg, the dose was 10 mg/kg.  - placebo  The drug was administered every 4 weeks for 24 weeks for a total of 7 doses.  Follow-up: 52 weeks | There was no difference in the 2-hour C-peptide mAUC at week 52 between the tocilizumab- and placebo-treated groups in either the pediatric or adult cohort  No significant differences were seen between treatment arms with respect to average total daily insulin usage or HbA1c in either the pediatric or adult cohort. | Mixed sample  (adults and children) |
| **Antigen-specific therapies** | | | | | |
| Walter M et al, 2009  [90] | Randomized, four-arm, placebo-controlled | n. 188 patients (age 10-35 y) with recently diagnosed T1D. | Randomization to s.c. administration of placebo or 1, 0.5, or 0.1 mg NBI-6024 at baseline, weeks 2 and 4, and then monthly until month 24  Follow-up: 24 months | The mean peak C-peptide concentration at 24 months after study entry showed no significant difference between the groups treated with 0.1 mg (0.59 pmol/ml), 0.5 mg (0.57 pmol/ml), and 1.0 mg NBI-6024 (0.48 pmol/ml) and the placebo group (0.54 pmol/ml). Fasting, stimulated peak, and AUC C-peptide concentrations declined linearly in all groups by approximately 60% over the 24-month treatment period. The average daily insulin needs at month 24 were also comparable between the four groups. No treatment-related changes in islet antibodies and T cell numbers were observed. | Mixed sample  (adults and children) |
| Ludvigsson J et al, 2012  [91] | Multicenter, double-blind, RCT | n. 327 patients with recent onset T1D and GAD65+, a fasting C-peptide level above 0.3 ng/mL, duration of T1D < 3 months.  n. 111 received 4 doses of GAD-alum (age 12.9 ± 2.4 y)  n. 108 2 doses of GAD-alum (age 12.9 ± 2.1 y)  n. 115 received placebo (age 13.3 ± 2.3 y) | Randomization performed in balanced blocks of six in:  -s.c. injections of 20 μg of GAD-alum on days 1, 30, 90, and 270 (four-dose regimen);  -s.c. injections of GAD-alum on days 1 and 30 and of placebo on days 90 and 270 (two-dose regimen);  -s.c. injections of placebo on days 1, 30, 90, and 270.  Follow-up: 15 months | Stimulated C-peptide levels showed a progressive decline from baseline to month 15 in all three study groups. At 15 months, the treatment effect of the 4-dose and 2-dose regimens combined was not significantly larger than the effect of placebo (P = 0.10)  The use of GAD-alum as compared with placebo did not affect the insulin dose, HbA1c level, or hypoglycemia rate. Adverse events were infrequent and mild in the three groups, with no significant differences. |  |
| Casas R et al, 2020  [92] | Single center open-labeled pilot clinical trial | n. 12 children and youth (age 12.6-23.1 y) with T1D from less than 180 days with fasting C-peptide ≥ 0.12 nmol/L and GAD65 ab+ | 4 μg GAD-alum into lymph-node at day 30, 60, and 90, and oral Vitamin D 2000 U/d, days 1 to 120  Follow-up: 15 months | At 15 month fasting C-peptide and insulin requirement remained stable while HbA1c was lower. Stimulated C-peptide showed no change at 6 months but declined after 15 months (81% of baseline).  11 patients remained in partial remission.  Patients with poorer clinical response had higher baseline levels of GAD65-induced cytokines and T-cell activation, and an increased ratio of effector/central memory T cells. | Small sample size  No randomization to placebo |
| Ludvigsson J et al, 2021  [93] | Randomized, placebo-controlled, double-blind trial | n. 109 patients (mean age 16.4 ± 4.1 y) with T1D and elevated serum GAD65 ab+ | Randomization to three intralymphatic injections (1 month apart) with 4 μg GAD-alum and oral vitamin D (2,000 IE daily for 120 days) or placebo  Follow-up: 15 months | GAD-alum–treated patients carrying HLA DR3-DQ2 (n = 29; defined as DRB1*03, DQB1*02:01) showed greater preservation of C-peptide AUC (treatment effect ratio 1.557 [CI 1.126–2.153]; P = 0.0078) after 15 months compared with individuals receiving placebo with the same genotype (n = 17) | The number of patients in the specified subgroup was smaller |
| Martin A et al, 2022  [94] | Single-center, double-blind randomized trial | n. 97 children with recent-onset T1D  - oral GABA: mean age 11.2 ± 3.9 y  - oral GABA plus two-doses GAD-alum: mean age 11.6 ± 3.2 y  - placebo: mean age 11.1 ± 3.5 y | Using a 2:1 treatment:placebo ratio, interventions included oral GABA twice-daily (n = 41), or oral GABA plus two-doses GAD-alum (n = 25), versus placebo (n = 31)  Follow-up: 12 months | The primary outcome, preservation of fasting/meal-stimulated c-peptide, was not attained. Of the secondary outcomes, the combination GABA/GAD reduced fasting and meal-stimulated serum glucagon, while the safety/tolerability of GABA was confirmed. There were no clinically significant differences in glycemic control or diabetes antibody titers. |  |
| **Other immunomodulant drugs** | | | | | |
| Bender C et al, 2024  [95] | Phase 2 RCT | n. 110 children (age 8-18 y) with newly diagnosed T1D | A single infusion of Autologous Polyclonal CD4+CD25+CD127lo/-FOXP3+ Regulatory T-cells [Tregs])  Randomization 1:1:1  - Low dose 1x10^6^ cells/Kg  - High dose 20 x 10^6^ cells/Kg  - Matching placebo  Follow-up: 12 months | The single dose of Tregs did not prevent decline in residual beta-cells function over 1 year compared to placebo (P = 0.94 low dose, P = 0.21 high dose).  No significant difference in stimulated C-peptide AUC at the primary end point. |  |
| Zielinski M et al, 2022  [96] | Phase 2 RCT | n. 35 children with newly diagnosed T1D  n. 13 Tregs only  n. 12 Tregs + rituximab  n. 11 control | Randomization 1:1:1 ratio  - Tr: autologous expanded CD3+CD4+CD25highCD127- Tregs in the two doses (30x10^6^ of Tregs/kg b.w. each), three months apart  - TrCD20: 4 doses of rituximab (375 mg/m2, each) between the first and second dose of Tregs  - Control group: no treatment  Follow-up: 24 months | At month 24, as compared with the control, both treatment groups remained superior in the C-peptide AUC at mixed meal tolerance test. The proportion of patients in remission was significantly higher in the combined group than in the control group at 3, 6, 9 and 21 months but not at 18 and 24 months. There was no significant difference between the Tregs only group and control group. | Small sample size |
| Haller MJ et al, 2018  [98] | Phase 2b  Placebo-controlled, three-arm, double-masked RCT | n. 89 participants with recent onset of T1D from the TrialNet Study  with at least 1 T1D-related autoantibody and C-peptide levels ≥0.2 nmol/L during a MMTT test  n. 29 ATG (age 17.2 ± 5.0 y)  n. 29 ATG alone (age 18.1 ± 6.9 y)  n. 31 placebo (age 16.9 ± 4.6 y) | Randomization 1:1:1 ratio to:  - ATG (2.5 mg/kg iv) followed by pegylated G-CSF (6 mg s.c. every 2 weeks for 6 doses)  - ATG alone (2.5 mg/kg)  - placebo  Follow-up: 12 months | At 12 months, the mean AUC C-peptide was significantly higher in subjects treated with ATG (0.646 nmol/L) versus placebo (0.406 nmol/L) (P = 0.0003) but not in those treated with ATG/G-CSF (0.528 nmol/L) versus placebo (P = 0.031).  HbA1c was significantly reduced at 1 year in subjects treated with ATG and ATG/GCSF, P = 0.002 and 0.011, respectively.  There were no differences in insulin use between either experimental treatment group or the placebo group | Small sample size  Mixed sample  (adults and children) |
| Haller MJ et al, 2016  [99] | Placebo-controlled single-blinded RCT | n. 25 participants (age 12-45 y) with established T1D (duration 4-24 months) and minimum peak C-peptide of 0.1 nmol/mL following a 4-h MMTT  n. 17ATG and pegylated G-CSF  n. 8 placebo | Randomization 2:1 ratio to:  - ATG (Thymoglobulin; Sanofi) (0.5 mg/kg on day 1 and 2 mg/kg on day 2 iv infusion for total dose of 2.5 mg/kg) and pegylated G-CSF (Neulasta; Amgen) (6 mg s.c. injection administered every 2 weeks for 6 doses)  - placebo  Follow-up: 24 months | At 24 months, MMTT-stimulated AUC C-peptide was not significantly different in ATG+G-CSF group (0.49 nmol/L/min) vs placebo (0.29 nmol/L/min). | Small sample size  Mixed sample  (adults and children) |
| Lin A et al, 2021  [100] | Placebo-controlled, single-blinded RCT | n. 25 patients (age 12–45 y) with established T1D (from 4 months to 2 years) | Randomization 2:1 to:  - iv low-dose ATG (2.5 mg/kg total dose over 2 days) and s.c. pegylated G-CSF (6 mg every 2 weeks for six doses)  - placebo  Follow-up: 5 years | After 5 years, there were no statistically significant differences in the C-peptide AUC when comparing patients who received ATG/GCSF versus placebo (P = 0.41). A model based on the mean trajectories of C-peptide AUC over 5 years was applied to recategorize responders (n = 9) and non-responders (n = 7), taking into account the different trends between groups. Responders to ATG/GCSF demonstrated a nearly unchanged HbA1c over the 5 years (mean [95% CI] adjusted change 0.29% [-0.69%, 1.27%]), but the study was not powered for comparison with non-responders 1.75% (-0.57%, 4.06%) or placebo recipients 1.44% (0.21%, 2.66%). | Small sample size,  Mixed sample  (adults and children) |
| Forlenza GP et al, 2023  [102] | Placebo-controlled double-blind,  multicenter  RCT | n. 88 patients (age 7-17 y) with T1D diagnosed within 31 days with at least 1+ Iab; weight 30 kg or greater  n. 47 verapamil  n. 41 placebo | Randomization 1:1 to:  - once-daily oral verapamil (dose dependent on each participant’s weight and started with 60 mg/d or 120 mg/d. The dose was escalated at 2- to 4-week intervals to a maximum of dose of 360 mg/d for participants weighing more than 50 kg)  -placebo    Follow-up: 52 weeks | In the verapamil group the mean C-peptide AUC was 0.66 pmol/mL at baseline and 0.65 pmol/mL at 52 weeks from diagnosis compared with 0.60 pmol/mL at baseline and 0.44 pmol/mL at 52 weeks in the placebo group. The adjusted between-group treatment difference at 52 weeks was 0.14 pmol/mL (95% CI, 0.01 to 0.27 pmol/mL; P = 0.04), representing a 30% higher C-peptide level with verapamil than with placebo.  During the 52-week MMTT, the mean peak C-peptide level was 0.83 pmol/mL (SD, 0.37 pmol/mL) in the verapamil group compared with 0.55 pmol/mL (SD, 0.34 pmol/mL) in the placebo group. The 52-week peak C-peptide level was 0.2 pmol/mL or greater in 41 of 43 participants (95%) in the verapamil group compared with 27 of 38 participants (71%) in the placebo group. | Small sample size  Limitation regarding commercially available dosing options for extended-release verapamil |
| Waibel M et al, 2023  [107] | Phase 2, placebo-controlled, double-blind RCT | n. 91 patients with a diagnosis of T1D within 100 days; the presence of at least 1 IAb+, and a random C-peptide level > 0.3 nmol/L or a C-peptide level >0.2 nmol/L during a 2-hour MMTT  n. 60 baricitinib (age 18.5±5.7 y)  n. 31 placebo (age 18.7±5.9 y) | Randomization 2:1 to:  baricitinib (4 mg/d) or  placebo orally for 48 weeks  Follow-up: 48 weeks | At the week 48, the median of the MMTT mean C-peptide level was 0.65 nmol/L/min (IQR, 0.31 to 0.82) in the baricitinib group and 0.43 nmol/L/min (IQR, 0.13 to 0.63) in the placebo group (adjusted mean difference in the ln[AUC+1], 0.13; 95% CI, 0.06 to 0.20; P = 0.001). The mean daily insulin dose at 48 was 0.41 U/kg/d (95% CI, 0.35 to 0.48) in the baricitinib group and 0.52 U/kg/d (95% CI, 0.44 to 0.60) in the placebo group. The levels of A1c were similar in the two trial groups.  The mean coefficient of variation of the glucose level as measured by CGM, was 29.6% (95% CI, 27.8 to 31.3) in the baricitinib group and 33.8% (95% CI, 31.5 to 36.2) in the placebo group.  The frequency and severity of adverse events were similar in the two trial groups, and no serious adverse events were attributed to baricitinib or placebo. | Mixed sample  (adults and children)  Small sample size  Exclusively Caucasians,  No patients younger than 10 years of age were enrolled |

Abbreviations: ATG = Anti-thymocyte globulin; AUC = area under the curve; CGM = continuous glucose monitoring, CI = confidence interval; d = day; FDR = first-degree relatives; IAb(s) = islet autoantibody(s); i.m. = intramuscular; iv = intravenous; MMTT = mixed-meal tolerance test; s.c. = subcutaneous, T1D = type 1 diabetes; y = years.
